# Protective effect of royal jelly on fertility and biochemical parameters in bleomycin-‎induced male rats

**Published:** 2014-03

**Authors:** Tayebeh Amirshahi, Gholamreza Najafi, Vahid Nejati

**Affiliations:** 1 *Department of Histology and Embryology, Faculty of Sciences, Urmia University, Urmia, Iran.*; 2 *Department of Anatomy and Embryology, Faculty of Veterinary Medicine, Urmia University, Urmia, Iran.*

**Keywords:** *Bleomycin*, *Royal jelly*, *Testis*, *Testosterone*, *Rat*

## Abstract

**Background:** Bleomycin (BL) is a glycopeptide antibiotic obtained from the bacterium Streptomyces verticillus which is routinely used for treatment of human cancers. Royal jelly (RJ) is a production from the hypo pharyngeal, mandibular and post cerebral glands of nurse bees. RJ consists of 66% water, 15% sugars, 5% lipids, and 13% proteins, essential amino acids and vitamins.

**Objective:** The aim of present study was to evaluate protective effect of royal jelly on sperm parameters and malondialdehyde (MDA) production in rat.

**Materials and Methods: **Forty adult male wistar rats (220±20gr) were randomly divided into 4 groups (n=10). Control group (CG) received normal saline 10 ml/kg twice a week with Intraperitoneal (I.P) for 48 days (0.3 ml/rat(. Royal Jelly group (RJG) received jelly (100 mg/kg daily) for 48 days orally. Bleomycin group (BLG) received BL (10 mg/kg twice a week) with I.P for 48 days. Royal Jelly+ Bleomycin group (RJ+BLG) received royal Jelly (100 mg/kg /day) orally concomitant with BL administration. Sperm count, motility, and viability were investigated and chromatin quality and DNA integrity were also analyzed. Serum testosterone and MDA concentrations were measured as well.

**Results: **BL caused decline significantly (p<0.05) sperm count, sperm viability, motility as well as testosterone concentration compared to control group while significant (p<0.05) increases in immature sperm, sperm with damaged DNA and MDA concentration were announced in BL in comparison with CG and RJ+BLG. Royal jelly improved Bleomycin-induced toxicity on sperm parameters and testosterone and MDA concentrations.

**Conclusion:** The present results support the idea that BL adversely affects sperm parameters and MDA and the RJ with antioxidant properties has positive effects on these parameters.

This article extracted from M.Sc. thesis. (Tayebeh amirshahi)

## Introduction

Bleomycin is a chemotherapic antibody produced by the bacterium Streptomyces verticillus. This antibiotic is an anti-tumor that plays an important role in the treatment of lymphomas, carcinomas and germ cells and makes its affect through creating single or double stranded DNA in tumor cells and interrupting the cell cycle (1). Currently the most common cancer affecting men in the reproductive age is testicular cancer (2). However, if diagnosed early, the disease is curable. Simultaneous administration of Bleomycin, Eutoposide and Cisplatin increases survival of patients with this cancer up to 5 years (3). 

Bleomycin may have a negative impact on reproductive function, fertility, and on children who have been treated (4). When used as an anticancer agent, the chemotherapeutical forms are primarily Bleomycin A2 and B2, which work by causing breaks in DNA. The drug is used in the treatment of Hodgkin’s lymphoma, squamous cell carcinomas, and testicular cancer, as well as in the treatment of plantar warts (5). According to current statistics, 35% of infertility cases in couples are related to males. A healthy man gives out 120-600 million sperm per ejaculation (6). More than 90% of male infertility is due to low sperm count, poor sperm quality, or both. The remaining cases of male infertility are caused by a wide range of anatomical problems, hormonal imbalances, and genetic defects. Royal Jelly (RJ), a principal food of the honeybee queen, is produced by the hypo-pharyngeal glands of worker honeybee and consists of 66% water, 15% sugars, 5% lipids and 13% proteins, essential amino acids and vitamins (7). 

It has been reported that RJ has pharmacological activities such as vasodilative and hypotensive activity, antitumor, anti-inflammatory, antihypertensive, anti-fatigue and anti-allergy activity (8, 9). RJ makes shorter the healing period of desquamated skin lesions (10). The Queen can survive up to 5 or 6 years using the jelly, while the worker bees live between 7-8 weeks (10). RJ is rich in minerals, natural hormones, vitamin B, essential fatty acids, with Molik acid and Aspartic acid that are important for tissue repair and growth (7). Therefore, in this study, we attempted to determine the effects of BL on sperm characteristics, serum testosterone levels, and biochemical changes related to oxidative stress in testes and to examine the protective effect of RJ on these parameters. 

## Materials and methods

**Animals**

In present study, 40 adult male Wistar rats (220±20 gr) were obtained from animal house of faculty of science, Urmia University, The rats were maintained under controlled room temperature of 22±3^o^C with 12 hours light/dark cycles and the humidity level of 50-60%. All animals had access to laboratory chow and tap water. This study was carried out following approval from the Ethical Committee on the use and care of experimental animals. This research is an experimental study.

**Drug treatment**

The animal were divided into 4 groups; each group was consisted of 10 rats.

Control group (CG) received saline 10 ml/ kg twice a week with I.P for 48 days (0.3ml/rat).

Royal Jelly group (RJG) received RJ at dose of 100 mg/kg daily for 48 days orally(27).

Bleomycin Group (BLG) the rats received BL at dose of 10 mg/kg twice a week with I.P for 48 days (11, 12).

RJ+ BLG received 10 mg/kg twice a week BL and100 mg/kg/day RJ for 48 days.

**Sperm characteristics**

**Sperm collection: **After 48 days, animals were anesthetized by Chloroform and sacrificed by dislocation of cervical vertebra. Tail of epididymis from each animal was finely minced and transferred into 1ml mR1ECM medium (medium for 1-cell rat embryos, mR1ECM) + 4mg/ml bovine serum albumin and incubating for 1 hours at 37^o^C in an atmosphere of 5% CO_2_ to allow sperm to swim up. 

**Sperm count: **The epididymal sperm count was determined by hemocytometry (Neubauer chamber) and the method described in the WHO manual (1999) (13). 5 micro liter aliquot of epididymal sperm was diluted with 95 micro liter of diluent (0.35% formalin containing 5% NaHCO_3_ and 0.25% trypan blue). A few drops of the diluted sperm suspension as a sample, was transferred into a Neubauer’s improved counting chamber (depth 0.1 mm), and allowed to stand for 5 min. The sperm heads were counted and expressed as million/ml of suspension.

**Sperm viability: **Sperm viability was evaluated as follows. 20 µl of 0.05% eosin Y and nigrosin were added into an equal volume of the sperm suspension. After 2 min of incubation at room temperature, slides were viewed by light microscope with magnification of 400×. Dead sperm cells appeared to be pink and live sperm cells were not stained. In each sample 400 sperm cells were counted and viability percentages were calculated (14).

**Sperm Motility: **In order to observe mobility, 10 microliters of semen was placed on a glass slide and covered with a lamella. Using a light microscope with a magnification of 400×, the number of sperm with rapid progressive forward movement (RPFM), slowly progressive forward movement (SPFM), Non-progressive motility ‏ (NPM) and motionless (ML) sperm cells were counted in several microscopic field of vision and percentage of motile and Immobile sperm cells was obtained (13).

**Sperm DNA integrity assays**

**Acridine-orange, DNA denaturation assay: **Acridine orange test (AOT) is a simple microscopic procedure based on acid conditions to denaturant DNA followed by staining with acridine orange. The AOT measures the metachromatic shift of AO fluorescence from green (native DNA) to red (denatured DNA). Acridine Orange fluoresces green when it binds to native DNA and red when it binds to the fragmented DNA. AOT using fluorescence microscopy provides a general picture of the status of DNA denaturation (15). 

AO intercalates into native DNA and the dye fluoresces green when exposed to blue light and red light when bound to single- stranded DNA. Thick smears were placed in Carnoy’s fixative (methanol: acetic acid 3:1) for 2h to fixation. After staining for 5 min the slides were rinsed with deionized water. Under the fluorescent light microscope, red and green sperms could be observed (100 sperm per slide were analyzed). 

**Aniline blue, chromatin quality assay: **Histones are replaced by transition proteins and then by Protamines during the later stages of spermatogenesis, spermatid nuclear changing and condensing. The DNA strands are tightly wrapped around the Protamines molecules to create toroidal structures (16). Protamines-rich nuclei of mature spermatozoa are rich in arginine and cysteine and contain relatively low levels of lysine, therefore they could not be stained by aniline blue (AB). Slides were prepared by smearing 5 μl of either a raw or washed semen sample. 

The slides are air-dried and fixed for 30 min in 3% glutaraldehyde in phosphate buffered saline. The smear was dried and stained for 5 min in 5% aqueous aniline blue solution (pH=3.5). Sperm heads containing immature nuclear chromatin stain blue and those with mature nuclei do not take up the stain. The percentage of spermatozoa stained with aniline blue was determined by counting 400 spermatozoa per slide under bright field microscope (17).

**Biochemical assays**

**Sample collection: **Blood samples were collected from the animals only at end of the study. The animals were fasted for 6 hours before the collection of blood samples. The animals were anesthetized and blood was taken through heart. Blood was drawn from all 10 animals in each group and centrifuged at 3000g for 10 minutes. Serum was separated and stored at -70^o^C until analysis. Testes were removed, cleared from adhering connective tissue. And were stored at -70^o^C until biochemical analysis. 

**Measurement of malondialdehyde level: **For this purpose, one gram of testicular tissue was Homogenized in 0.05 M phosphate buffer at pH=7.4 and the concentration of 10% (w/v). Then the obtained solutions were centrifuged in1000g and supernatants were used for the evaluation of levels of lipid peroxidation products. Levels of lipid peroxidation were measured using cheeseman and Esterbauer method (18). On the degree of malondialdehyde (MDA) production, lipid peroxidation can be determined. Malondialdehyde is the final product of fatty acid peroxidation and reacts with Thiobarbituric acid (TBA) and creates a colorful complex.

Spectrophotometric measurement method is based on the color produced by reaction of TBA with MDA. For this purpose, 300 ml of tri-chloric acid 10% was added to 150 Microliter supernatant of centrifuged sample, and then centrifuged for 10 min at 4^o^C and 1000g. 300 microliters of supernatant were transferred to a test tube and was incubated with 300 microliters of thiobarbituric acid 0.67% at 100^o^C for 25 min. 5 min after cooling the solution, the pink color due to the reaction of TBA-MDA appeared and was measured using a spectrophotometer at a wavelength of 535 nm. Concentration of MDA was calculated using the coefficient of TBA-MDA complex absorption and was expressed as nmol/g wet tissue (19).

**Testosterone assay: **After blood sampling, the serum was separated using a centrifuge (3000 gr for 6 min) and kept at -70^o^C until analysis of testosterone hormone. Serum testosterone concentrations were measured by using immune-radiometric technique using the kits of WHO/Sigma Asso-RTGC-768/98. 

**Statistical analysis**

The data are presented as the mean±SE. Differences between groups were analyzed by ANOVA followed by Tukey test using SPSS package, version 16 and level of significance was taken as p<0.05.

## Results

In present study, data revealed that sperm count and sperm viability in BL group decreased significantly in comparison with control and RJ groups (p<0.05). RJ co-administrated with BL announced partially improvement and enhancement in number of sperm and sperm viability that were presented in Table I. There was no significant (p<0.05) value in sperm maturity among mentioned groups. Furthermore, in comparison to the control and royal jelly groups, were increased sperm cells with DNA damage in BL group. The rats were administrated RJ simultaneously BL demonstrated a decline in sperm DNA damage (Table II). Our investigation on sperm motility revealed that RPFM was decreased significantly (p<0.05) in BL group in comparison to the CG and RJ. Percentage of SPFM and NPM sperm cells as well as ML was increased in BL group. This study announced that administration of RJ simultaneously with BL caused a partially increase in the sperm motility type SPFM in comparison with BL. 

**Testosterone assay**

In this study, according to table 3, it is specified that the testosterone concentration in the BL group shows significant (p<0.05) reduction with CG and RJ groups. In RJ-BL group revealed remarkable increased (p<0.05) testosterone level in blood circulation in comparison to BL group. 

**MDA assay**

BL caused induced lipid per oxidation in the tissue of testis as demonstrated by significant (p<0.05) elevation of MDA in BL to compare with CG and RG. MDA level in RJ-BL were lower those in the BL. RJ administration caused significantly decline of MDA in RJ-BL group.

**Table I:** The effect of bleomycin and Royal jelly on Sperm parameters in experimental groups

**Group**	**Sperm count** **(×10** ^6^ **/ml)**	**Sperm viability** **(%)**	**Sperm motility** **(RPFM) (%)**	**Sperm motility** **(SPFM) (%)**	**Sperm motility** **(NPM) (%)**	**Sperm motility** **(ML) (%)**
Control (CG)	37.36 ± 1.15	86.96 ± 0.90	63.50 ± 1.18	17.53 ± 0.67	11.23 ± 0.60	7.73 ± 0.37
Royal jelly (RJ)	38.80 ± 1.53	86.80 ± 2.28	62.43 ± 1.15	16.83 ± 0.33	11.13 ± 0.58	10.60 ± 0.30
Bleomycin (BL)	25.77±0.82*^a^^b^	67.46 ± 1.24*^a^^b^	51.63 ± 0.50*^a^	23.86 ± 0.66*^a^^b^	12.03 ± 0.52	12.46 ± 0.73
Royaljelly+Bleomycin (RJ+BL)	34.70 ±2.42*^c^	84.40 ± 2.66*^c^	59.80 ± 2.36*^c^	16.13 ± 2.03*^c^	12.50 ± 1.50	11.56 ± 0.58

Data are presented as Mean±S.E .

* p<0.05.

a : significant compared with control.

b : significant compared with RJG.

c : significant compared with BLG.

**Table II:** The effect of bleomycin and Royal jelly on sperm maturity and sperm DNA damage in rat

**Group**	**AO+ (%)**	**AB+ (%)**
Control (CG)	12.66 ± 0.27	6.36 ± 0.52
Royal jelly (RJ)	10.36 ± 0.58	7.30 ± 0.43
Bleomycin (BL)	20.30 ± 0.52*^a^^b^	11.10 ± 0.87*^a^^b^
Royal jelly+ Bleomycin (RJ+BL)	17.43 ± 0.49*^c^	9.36 ± 075*^c^

AO+: Acridine-orange positive (sperm with DNA damage). AB+: Aniline blue positive (immature sperm)

Data are presented as Mean±S.E.

* p<0.05.

a : significant compared with control.

b : significant compared with RJG.

c : significant compared with BLG.

**Table III:** The effect of bleomycin and Royal jelly on testis lipid peroxidation and testosterone production in rat

**Group**	**MDA (µmol/gr tissue)**	**Testosterone (ng/ml)**
Control (CG)	484.75 ± 16.86	4.28 ± 0.23
Royal jelly (RJ)	461.90 ± 10.52*^c^	4.10 ± 0.26
Bleomycin (BL)	643.14 ± 5.05*^a^^b^	2.47 ± 0.17*^a^^b^
Royal jelly+ Bleomycin (RJ+BL)	514.48 ± 16.90*^c^	3.84 ± 0.08*^c^

Data are presented as Mean±S.E .

* p<0.05.

a : significant compared with control.

b : significant compared with RJG.

c : significant compared with BLG

**Figure 1:**
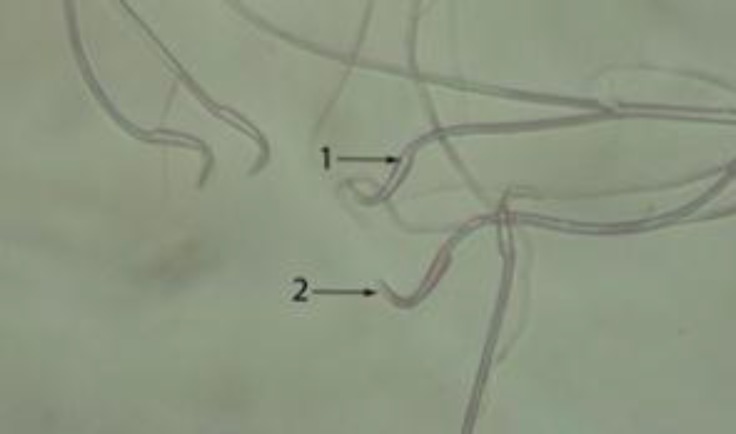
Live sperm (1) with uncoloured head and dead sperm with pink or red (2) head in control group (E&N ×400).

**Figure 2:**
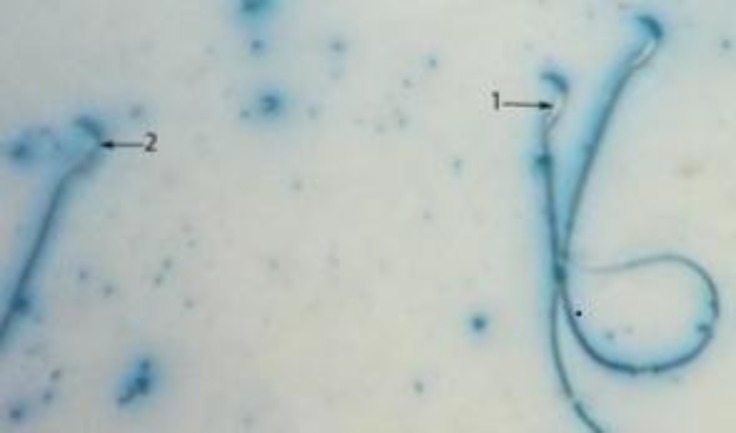
Sperm head with mature nuclei is light blue (1) and Sperm head containing immature nuclear chromatin is dark blue (2) (AB ×400).

**Figure 3:**
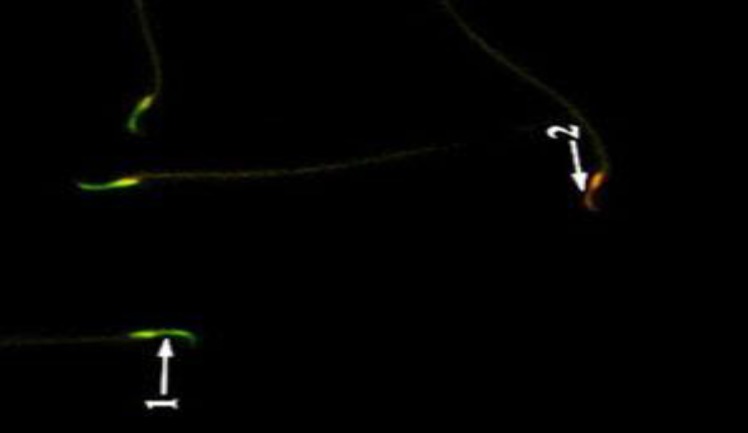
Sperm with normal DNA integrity is seen green color (1) and Sperm cells with orange color (2) head which have damaged chromatin in RJ group (AO ×400).

## Discussion

During the last 50 years, chemotherapy techniques are used to prevent diseases such as testicular cancer. In this way, certain chemical drugs are prescribed. Although taking these drugs has increased the survival rate of patients and the recovery, But are associated with serious side effects. These side effects can be unpleasant changes to the body’s normal function (4). In this study, it will be discussed whether Bleomycin, an anti- testicular cancer antibiotic, can affect sperm function, chromatin integrity, sperm motility, sperm count and whether royal jelly can improve Bleomycin-induced damage. Sperm count reduction is an important indicator of male infertility (20).

Change in the germ cell function can alter sperm count. Any agent that interferes with mitotic division is also known to reduce the sperm count (21). O’Flaherty *et al* in 2010 showed that sperm parameters, hormone levels and testicular volume in survivors of testicular cancer is quite different from normal people that these people are at risk of having an abnormal reproduction (22). Our studies showed that Bleomycin greatly reduced the percentage of sperm viability and sperm count compared to the control group. However, these parameters were much greater in the group treated with royal jelly. 

Cisplatin treatment causes an increase in lipid peroxide levels and a decrease in the activities of antioxidant enzymes that protect against lipid peroxidation in the tissues such as liver, kidney and testes (23, 24). Many cellular pathways have been suggested to contribute to induction of a state of oxidative stress and lipid peroxides. For example, it is possible that BL-induced oxidative stress and cytochrome P450 2E1-(CYP2E1-) mediated oxidative stress synergize to produce hepatotoxicity (25). The present study indicated that lipid peroxidation (MDA) in testis significantly increased in rats treated with BL alone. This result agrees with previous studies which have demonstrated the involvement of oxidative stress and lipid peroxidation in cis-induced liver and testes toxicities (26-30). 

Testis MDA levels significantly was improved with the Prophylactic RJ treatment. The RJ contains amino acids such as aspartic acid, cysteine, cystine, tyrosine, glycine, lysine, leucine, valine, and isoleucine that are biologically active. The antioxidant effect of RJ may be related to its free amino acids content, which has been indicated by previous researchers (31). Nuclear DNA integrity and the composition of chromatin proteins are important for normal sperm function (32). The process of spermatogenesis, in which histones are replaced first by transition proteins and then by Protamines, resulting in a very condensed sperm DNA structure, is absolutely critical for sperm chromatin packaging (33). 

Only a small percentage of the sperm genome remains associated with histones in mature sperm; this is approximately 15% in human spermatozoa and only 1-2% in rodent spermatozoa (34, 35). Recent findings have showed that the particular fashion in which residual histones in sperm nuclei are not arbitrary but appears to be organized in a way that their association with promoters of key gene regulators will be preferentially maintained (36). Abnormal histone retention or protamines deficiency in sperm is in association with the reduction in fertility and increased risk of embryonic failure after fertilization (37, 38). 

Besides protamines and residual histones, other sperm head proteins may be in close association with sperm chromatin and play a crucial role in normal sperm function. Packaging of sperm chromatin serves in reprogramming the paternal genome and sets the appropriate genes ‎to be expressed in the early stages of embryo development (33). Therefore, a correct chromatin ‎packaging level will be essential to fully express the fertilizing capacity of sperm. Abnormal nucleoprotein ‎content and/or DNA strand breaks‎ can be related to defects of sperm chromatin, which can be evaluated ‎using ‎staining techniques such as chromomycin A3‎ or acridine orange, the later method we used in our ‎study (39). This study showed that Bleomycin will adversely affect sperm chromosomes while less chromosomal ‎damage was observed in the royal jelly treated group. 

Acridine Orange staining showed that in Group 3, ‎the amount of denatured DNA is much greater than the control group. These rates were lower in group ‎‎4 treated with royal jelly. Kamiguchi *et al* examined effects of five chemical drugs in terms of ‎toxicity on testicular tissue (40). In order to evaluate the testes function, testosterone and progesterone ‎levels were measured. They showed ketoconazole and aminoglutethimide inhibit secretion of ‎testosterone and progesterone (41). Spironolactone blocks testosterone secretion and increases ‎progesterone concentrations. Chlorpromazine inhibits the secretion of gonadotropins, testosterone, and ‎progesterone, which is a significant cytotoxic effects produced by these drugs (40‎‏,‏‎ 41).

Lambert injected thymidine, cyclophosphamide, chlorambucil, Theo TEPA, CCNU-busulphan and Procarbacine compounds to rats. These substances caused damage to the testicles, reduced testicles thymidine and increased its level in patient's serum. Actinomycin D and Vinblastin cause changes in the mixture of testicles thymidine. These chemical agents disrupt DNA synthesis (42). 

Silici *et al* in 2010 investigated the effect of royal jelly on cisplatin induced toxicity in the testis tissue. They stated that cisplatin has led to changes in sperm parameters and decreases fertility of individuals. They also stated that the use of cisplatin increases MDA and testicular tissue toxicity. They thought the increased levels of free radicals to be a factor for this. However, in their studies, royal jelly significantly reduced bleomycin-induced toxicity. Among these positive changes were reduced MDA level, improvement in testes tissue and sperm parameters (27). In the present study, testosterone levels in the treatment group was reduced significantly compared to the control group, but royal jelly significantly increased testosterone levels.

## Conclusion

In conclusion, the experimental results reveal that BL plays negative roles on reproductive system and function in sexually mature male rats by its adverse effects on sperm parameters and testosterone production in these animals. RJ as a complex, due to beneficial biological properties of its components, has been determined to exhibit antioxidant capacity. Therefore, RJ could help to prevent testicular toxicity manifested by BL chemotherapy. RJ has a possible protective effect against BL-induced spermiotoxicity effect when given after BL administration.

## References

[1] Du L, Sánchez C, Chen M, Edwards DJ, Shen B (2000). The biosynthetic gene cluster for the antitumor drug bleomycin from Streptomyces verticillus ATCC15003 supporting functional interactions between nonribosomal peptide synthetases and a polyketide synthase. Chem Biol.

[2] Bray F, Richiardi L, Ekbom A, Pukkala E, Cuninkova M, Moller H (2006). Trends in testicular cancer incidence and mortality in 22 European countries: continuing increases in incidence and declines in mortality. Int J Cancer.

[3] Robinson D, Moller H, Horwich A (2007). Mortality and incidence of second cancers following treatment for testicular cancer. Br J Cancer.

[4] Maselli J, Hales B, Chan P, Robaire B (2012). Exposure to Bleomycin, Etoposide, and Cis-Platinum Alters Rat Sperm Chromatin Integrity and Sperm Head Protein Profile12,3,5,6. Biol Reprod.

[5] Lewis TG, Nydorf ED (2006). Intralesional Bleomycin for warts: a review. J Drugs Dermatol.

[6] Adam G, Avise John C (2003). Male Pregnancy. Cur Biol.

[7] Sver L, Orsolic N, Tadic Z (1996). Royal jelly as a new potential immunomodulator in rats and mice. Comp Immunol Microbiol Infect Dis.

[8] Nagai T, Inoue R (2004). Preparation and the functional properties of water extract and alkaline extract of royal jelly. Food Chem.

[9] Inoue S, Koya-Miyata S, Ushio S, Iwaki K, Ikeda M, Kurimoto M (2003). Royal jelly prolongs the life span of C3H/HeJ mice: correlation with reduced DNA damage. Exp Gerontol.

[10] Hiroyuki M, Takahide I, Kazuo K, Kei F, Ichiro M, Hideyuki O (2012). Effect of royal jelly ingestion for six months on healthy volunteers. Nutr J.

[11] Arslan SO, Zerin M, Vural H (2002). The effect of melatonin on bleomycin-induced pulmonary fibrosis in rats. Coskun AJ Pineal Res.

[12] Yildirim Z, Kotuk M, Iraz M, Kuku I, Ulu R, Armutcu F (2005). Attenuation of bleomycin-induced lung fibrosis by oral sulfhydryl containing antioxidants in rats: erdosteine and Nacetylcysteine. Pulm Pharmacol Ther.

[13] ( 1999). WHO laboratory manual for the examination of human semen sperm-cervical mucus interaction.

[14] Wyrobek AJ, Gordon LA, Burkhart JG, Francis MW, Kapp RW, Letz G (1983). An evaluation of mouse sperm morphology test and other sperm tests in non-human mammals. Mutat Res.

[15] Varghese A, Fischer-Hammadeh C, Hammadeh M (2011). Acridine Orange Test for Assessment of Human Sperm DNA Integrity.

[16] Rezvanfar MA, Sadrkhanlou RA, Ahmadi A, Shojaei-Sadee H, Rezvanfar MA, Mohammadirad A (2008). Protection of cyclophosphamide-induced toxicity in reproductive tract histology, sperm characteristics, and DNA damage by an herbal source; evidence for role of free-radical toxic stress. Hum Exp Toxicol.

[17] Hammadeh M, Zeginiadov T, Rosenbaum P (2001). Predictive value of sperm chromatin condensation (aniline blue staining) in the assessment of male fertility. Arch Androl.

[18] Esterbauer H, Chesseman KH (1990). Determination of aldehydic lipid peroxidation product: malonaldhyde and 4-hydroxynonenal. Methods Enzymol.

[19] Hosseinzade H, Sadeghnia RH (2005). Safranal, aconstituent of corcus sativus (saffron), attenuated cerebral ischemia induced oxidative damage in rat hippocampus. J Pharm Pharm Sci.

[20] Kumar SG, Narayana K, Bairy KL, D'Souza UJ, Samuel VP, Gopalakrishna K (2006). Dacarbazine induces genotoxic and cytotoxic germ cell damage with concomitant decrease in testosterone and increase in lactate dehydrogenase concentration in the testis. Mutat Res.

[21] van der Spoel AC, Jeyakumar M, Butters TD, Charlton HM, Moore HD, Dwek RA (2002). Reversible infertility in male mice after oral administration of alkylated imino sugars: A nonhormonal approach to male contraception. Proc Natl Acad Sci USA.

[22] O'Flaherty C, Hales B, Chan P, Robaire B (2010). Impact of chemotherapeutics and advanced testicular cancer or Hodgkin lymphoma on sperm deoxyribonucleic acid integrity. Fertil Steril.

[23] Goldstein RS, Mayor GH (1983). Mini review the nephrotoxicity of Cisplatin. Life Sci.

[24] Cayir K, Karadeniz A, Yildirimetal A (2009). Protective effect of L-carnitine against cisplatin-induced liver and kidney oxidant injury in rats. Cent Eur J Med.

[25] Lieber CS (1997). Cytochrome P-4502E1: its physiological and pathological role. PhysiolRev.

[26] Favareto AP, de Toledo FC, Kempinas Wde G (2011). Paternal treatment with cisplatin impairs reproduction of adult male offspring in rats. Reprod Toxicol.

[27] Silici S, Ekmekcioglu O, Kanbur M, Deniz K (2010). The protective effect of royal jelly against cisplatin-induced renal oxidative stress in rats. World J Urol.

[28] Atessahin A, Yilmaz S, Karahan I, Ceribasi A, Karaoglu O (2005). Effects of lycopene against cisplatin-induced nephrotoxicity and oxidative stress in rats. Toxicology.

[29] Yapar K, Çavuşoǧlu K, Oruç E, Yalçin E (2009). Protective effect of royal jelly and green tea extracts effect against cisplatin-induced nephrotoxicity in mice: a comparative study. J Med Food.

[30] Iraz M, Ozerol E, Gulecetal M (2006). Protective effect of caffeic acid phenethyl ester (CAPE) administration on cisplatin-induced oxidative damage to liver in rat. J Cell Biochem Func.

[31] Tamura S, Kono T, Harada C, Yamaguchi K, Moriyama T (2009). Estimation and characterisation of major royal jelly proteins obtained from the honeybee Apismerifera. Food Chem.

[32] Delbes G, Hales BF, Robaire B (2010). Toxicants and human sperm chromatin integrity. Mol Hum Reprod.

[33] Braun RE (2001). Packaging paternal chromosomes with protamine. Nat Genet.

[34] Gatewood JM, Cook GR, Balhorn R, Bradbury EM, Schmid CW Sequence specific packaging of DNA in human sperm chromatin (1987). Science.

[35] Balhorn R, Gledhill BL, Wyrobek AJ (1977). Mouse sperm chromatin proteins: quantitative isolation and partial characterization. Biochemistry.

[36] Carrell DT, Hammoud SS (2010). The human sperm epigenome and its potential role in embryonic development. Mol Hum Reprod.

[37] Aoki VW, Liu L, Jones KP, Hatasaka HH, Gibson M, Peterson CM (2006). Sperm protamine 1/protamine 2 ratios are related to in vitro fertilization pregnancy rates and predictive of fertilization ability. Fertil Steril.

[38] Singleton S, Mudrak O, Morshedi M, Oehninger S, Zalenskaya I, Zalensky A Characterization of a human sperm cell subpopulation marked by the presence of the TSH2B histone (2007). Reprod Fertil Dev.

[39] Sergerie M, Laforest G, Bujan L, Bissonnette F, Bleau G (2005). Sperm DNA fragmentation: threshold value in male fertility. Hum Reprod.

[40] Kamiguchi Y, Tateno H (2002). Radiation- and chemical-induced structural chromosome aberrations in human spermatozoa. Mutat Res.

[41] Lambert B, Eriksson G (1979). Effects of cancer chemotherapeutic agents on testicular DNA synthesis in the rat. Evaluation of a short-term test for studies of the genetic toxicity of chemicals and drugs in vivo. Mutat Res.

[42] Brun HP, Leonard JF, Moronvalle V, Caillaud JM, Melcion C, Cordier A (1991). Pig Leydig cell culture: A useful in vitro test for evaluating the testicular toxicity of compounds. Toxicol Appl Pharmacol.

